# Factors influencing satisfaction with male circumcision in Taiwan

**DOI:** 10.1038/s41598-022-20140-8

**Published:** 2023-02-09

**Authors:** Chia Hung Chen, Wei-Ming Cheng, Yu-Hua Fan, Tung-Ping Chang

**Affiliations:** 1 Division of Urology, Department of Surgery, Taipei City Hospital, Zhongxiao Branch, Taipei, Taiwan; 2 Department of Urology, College of Medicine, National Yang Ming Chiao Tung University, Taipei, Taiwan; 3 Program in Molecular Medicine, College of Life Science, National Yang Ming Chiao Tung University, Taipei City, Taiwan; 4 Institute of Biopharmaceutical Science, National Yang Ming Chiao Tung University, Taipei, Taiwan; 5 Department of Urology, Taipei Veterans General Hospital, Taipei City, Taiwan; 6 Division of Urology, Department of Surgery, Taipei City Hospital, Renai Branch, No. 10, Sec. 4, Ren’ai Rd. Da’an Dist., Taipei City, 106243 Taiwan

**Keywords:** Urology, Urogenital diseases, Urological manifestations, Patient education, Public health

## Abstract

We aimed to investigate patient satisfaction with male circumcision in Taiwan. An online, questionnaire-based, cross-sectional study involving 376 circumcised men 20 to 40 years of age in Taiwan was conducted. Circumcision-related satisfaction was defined as a visual analogue scale score ≥ 6 (range, 1–10). Pearson’s chi-square test was performed to compare differences between satisfied and unsatisfied participants. Factors predictive of participant satisfaction were analysed using multivariate logistic regression. Statistical significance was set at *P* < 0.05. Among 376 circumcised men, 249 (66.2%) reported satisfaction with circumcision. Satisfied participants had higher levels of education, underwent circumcision for phimosis or balanitis, underwent circumcision during adulthood, reported a larger penile size, and had fewer long-term complaints. Furthermore, 89.4% had various long-term complaints, including skin colour mismatch, changes in masturbation methods, hypertrophic scarring, excessive shortening of the prepuce, and redundant prepuce. Multivariate analysis revealed that adult circumcision and the absence of long-term conditions were predictive of satisfaction. Two-thirds of participants were satisfied with their circumcision outcome, especially those who underwent circumcision for phimosis or balanitis during adulthood. Proper preoperative patient selection and postoperative symptom prevention would improve patient satisfaction.

## Introduction

Male circumcision, a common procedure worldwide, involves the removal of the foreskin of the penis^1^. Indications for this procedure include phimosis, dyspareunia, balanitis, and concurrent phimosis and balanitis^2^. The Centers for Disease Control and Prevention in the United States released recommendations promoting male circumcision as public health and preventive measure against sexually transmitted diseases^3^. The World Health Organization announced that voluntary medical male circumcision is effective for preventing human immunodeficiency virus infection; therefore, it recommended that men living in high-risk areas should undergo circumcision^4^. However, some males undergo circumcision for religious or cosmetic reasons. We hypothesised that because males have different reasons for circumcision, they also have different expectations regarding the outcome and that these expectations affect patient satisfaction. Understanding the predictive factors associated with postoperative satisfaction is crucial for patient selection and counselling before circumcision; however, there is limited information regarding the relationship between circumcision and patient satisfaction.

Moreover, there are several myths surrounding male circumcision, including the belief that the penis would become larger in size, and that the ejaculatory latency time would increase after circumcision. We investigated subjective perceptions of changes in penile length and ejaculatory latency time after circumcision as well as the prevalence of long-term complaints, such as skin colour mismatch, among circumcised men to facilitate appropriate patient selection and improve patient counselling.

## Results

A total of 376 men were included in this study. Of the participants, 249 (66.2%) reported satisfaction with the outcome of circumcision; however, the remaining 127 (33.8%) felt unsatisfied (Table 1). Phimosis and balanitis, cosmetic reasons or premature ejaculation, and being forced by their parents were reasons for circumcision for 220 (58.5%), 95 (25.3%), and 125 (33.2%) patients, respectively. After circumcision, 175 participants (46.5%) subjectively perceived that their penis had become longer, and 86 (22.9%) reported that they had a longer ejaculatory latency time. Nearly 90% of the participants reported some sort of long-term complaints, including skin colour mismatch (50.3%), changes in masturbation methods (33.0%), hypertrophic scarring (34.3%), excessive shortening of the prepuce (9.0%), and redundant prepuce (3.2%).

**Table 1:** Demographic data of factors influencing satisfaction.

Variables	Total (N = 376) (%)		Unsatisfied (N = 127) (%)		Satisfied^a^ (N = 249) (%)		*P* value
**Age at participation**							
≤ 30 years	183	48.70	61	48	122	49.00	0.86
> 30 years	193	51.30	66	52.00	127	51.00	
**Level of education**							
≤ Senior high school	63	16.80	28	22.00	35	14.10	0.05*
≥ College	313	83.20	99	78	214	85.90	
**Age at circumcision**							
Before adulthood^b^	215	57.20	84	66.10	131	52.60	0.012*
During adulthood	161	42.80	43	33.90	118	47.40	
**First sexual experience**							
No	55	14.60	17	13.40	38	15.30	0.865
≤ Senior high school	96	25.50	32	25.20	64	25.70	
≥ College	225	59.80	78	61.40	147	59.00	
**Circumcision after the first sexual experience**							
No	212	56.40	80	63.00	132	53.00	0.065
Yes	164	43.60	47	37.00	117	47.00	
**Sexual activity frequency**							
Monthly	25	6.60	9	7.10	16	6.40	0.779
Weekly	149	39.60	53	41.70	96	38.60	
Daily	202	53.70	65	51.20	137	67.80	
**Reason for circumcision**							
Forced by parents	125	33.20	52	40.90	73	29.30	0.024*
Phimosis and balanitis	220	58.50	64	50.40	156	62.70	0.023*
Cosmetic reasons and premature ejaculation	95	25.30	25	19.70	70	28.10	0.075
Others	52	13.80	19	15.00	33	13.30	0.65
**Penile length after circumcision**							
No change	201	53.50	91	71.70	110	44.20	0.0001*
Longer	175	46.50	36	28.30	139	55.80	
**Ejaculatory latency time**							
No change	290	77.10	99	78	191	76.70	0.786
Longer	86	22.90	28	22.00	58	23.30	
**Long-term complaints**							
No	40	10.60	7	5.50	33	13.30	0.021*
Yes	336	89.40	120	94.50	216	86.70	
Skin colour mismatch	189	50.30	80	63.00	109	43.80	0.0001*
Change in masturbation methods	124	33.00	41	32.30	83	33.30	0.838
Hypertrophic scarring	129	34.30	54	42.50	75	30.10	0.017*
Excessive shortening of prepuce	34	9.00	18	14.20	16	6.40	0.013*
Redundant prepuce	12	3.20	6	4.70	6	2.40	0.227

Chi-square test calculated for the difference in variables for satisfaction.

*P < 0.05.

^a^Visual analogue scale score ≥ 6 (range,1–10) is defined as satisfied.

^b^Adulthood means over 20 years old.

A higher percentage of satisfied circumcised men had a college degree (85.9% of satisfied participants versus 78.0% of unsatisfied participants; *P* = 0.05) and were circumcised during adulthood (47.4% versus 33.9%; *P* = 0.012). Circumcision for phimosis or balanitis was also associated with satisfaction (62.7% versus 50.4%; *P* = 0.023); however, men who were forced to undergo circumcision at the insistence of their parents tended to be unsatisfied (29.3% versus 40.9%; *P* = 0.024). A subjectively larger penile size after circumcision was significantly associated with satisfaction (55.8% versus 28.3%; *P* < 0.0001), but a longer ejaculatory latency time was not (23.0% versus 22.0%; *P* = 0.786). The presence of long-term complaints influenced the satisfaction with circumcision (86.7% versus 94.5%; *P* = 0.021), especially skin colour mismatch (43.8% versus 63.0%; *P* < 0.0001), hypertrophic scarring (30.1% versus 42.5%; *P* = 0.017), and excessive shortening of the prepuce (6.4% versus 14.2%; *P* = 0.013).

A univariate analysis revealed that circumcision during adulthood (odds ratio [OR], 1.76; 95% confidence interval [95% CI], 1.129–2.743; *P* = 0.013) was associated with satisfaction (Table 2). Circumcision for phimosis or balanitis (OR, 1.651; 95% CI, 1.072–2.544, *P* = 0.023) and not being forced by parents (OR, 1.672; 95% CI, 1.069–2.613; *P* = 0.024) were predictive of satisfaction; however, circumcision for cosmetic reasons or premature ejaculation was not predictive of satisfaction (OR, 1.596; 95% CI, 0.951–2.677; *P* = 0.077). The subjective perception of penile elongation after circumcision was associated with satisfaction (OR, 3.194; 95% CI, 2.017–5.059; *P* < 0.0001); however, prolonged ejaculatory latency time after circumcision was not associated with satisfaction (OR, 1.074; 95% CI, 0.643–1.792; *P* = 0.786). The presence of long-term complaints after circumcision influenced satisfaction (OR, 2.619; 95% CI, 1.124–6.101; *P* = 0.026), especially skin colour mismatch (OR, 2.186; 95% CI, 1.410–3.390; p < 0.0001), hypertrophic scarring (OR, 1.716; 95% CI, 1.101–2.675; *P* = 0.017), and excessive shortening of the prepuce (OR, 2.405; 95% CI, 1.181–4.895; *P* = 0.016).

**Table 2:** Comparative univariate and multivariate logistic regression analyses for the factors associated with satisfaction with circumcision.^a^

Factors associated the satisfaction with circumcision^b^	Univariate analysis			Multivariate analysis		
	OR	95% CI	*P* value	OR	95% CI	*P* value
**Age**						
≤ 30 years	(Ref)					
> 30 years	0.962	0.627–1.476	0.86			
**Level of education**						
≤ Senior high school	(Ref)			(Ref)		
≥ College	1.729	0.997–3.001	0.051	1.725	0.953 – 3.124	0.072
**Age at circumcision**						
Before adulthood^c^	(Ref)			(Ref)		
During adulthood	1.76	1.129 – 2.743	0.013*	1.771	1.008 – 3.112	0.047*
**First sexual experience**						
No	(Ref)					
≤ Senior high school	0.895	0.439–1.824	0.76			
≥ College	0.843	0.447–1.59	0.598			
**Circumcision after the first sexual experience**						
No	(Ref)			-		
Yes	1.509	0.974–2.337	0.066			
**Frequency of sexual activity**						
Monthly	(Ref)					
Weekly	1.019	0.421–2.463	0.967			
Daily	1.186	0.498–2.825	0.701			
**Reasons for circumcision**						
**Forced by parents**						
Yes	(Ref)			(Ref)		
No	1.672	1.069–2.613	0.024*	0.702	0.390–1.266	0.240
**Phimosis or balanitis**						
No	(Ref)			(Ref)		
Yes	1.651	1.072–2.544	0.023*	1.281	0.770–2.131	0.341
**Cosmetic reasons or premature ejaculation**						
No	(Ref)					
Yes	1.596	0.951–2.677	0.077			
**Skin colour mismatch**						
Yes	(Ref)			(Ref)		
No	2.186	1.410–3.390	< 0.0001*	2.543	1.591–4.065	< 0.0001*
**Change in masturbation methods**						
Yes	(Ref)					
No	0.953	0.604–1.504	0.838			
**Hypertrophic scarring**						
Yes	(Ref)			(Ref)		
No	1.716	1.101–2.675	0.017*	2.282	1.391–3.743	0.001*
**Excessive shortening of prepuce**						
Yes	(Ref)			(Ref)		
No	2.405	1.181–4.895	0.016*	3.118	1.433–6.784	0.004*
**Redundant prepuce**						
Yes	(Ref)					
No	2.008	0.634–6.358	0.236			

*OR* odds ratio, *CI* confidence interval.

*p < 0.05.

^a^Logistic regression test was calculated for the difference in factors associated with satisfaction with circumcision.

^b^Factors showed a difference in the univariate logistic regression test included in multivariate logistic regression. The level of education with *p* = 0.051 is closed to the definition of significance and showed a difference in the chi-square test, so we enrolled this factor in multivariate logistic regression.

^c^Adulthood means over 20 years old.

Based on the multivariate analysis, only circumcision during adulthood (OR, 1.771; 95% CI, 1.008–3.112; *P* = 0.047) and the absence of skin colour mismatch (OR, 2.543; 95% CI, 1.591–4.065; *P* < 0.0001), hypertrophic scarring (OR, 2.282; 95% CI, 1.391–3.743; *P* = 0.001), and excessive shortening of the prepuce (OR, 3.118; 95% CI, 1.433–6.784; *P* = 0.004) after circumcision were predictive of participant satisfaction. We also evaluated the effects of individual reasons for circumcision on participant satisfaction by controlling for the level of education, age at circumcision, and long-term complaints, including skin colour mismatch, hypertrophic scarring, and excessive shortening of the prepuce (Table 3). None of these reasons affected satisfaction with circumcision. However, circumcision during adulthood and the absence of long-term complaints remained predictive of satisfaction.

**Table 3:** Patient satisfaction based on reasons for circumcision after controlling for level of education, age at circumcision, and long-term complaints.

Satisfaction with circumcision	Forced by parents			Phimosis or balanitis			Cosmetic reasons or premature ejaculation		
	OR	95% CI	*P* value	OR	95% CI	*P* value	OR	95% CI	*P* value
**Level of education**									
≤ Senior high school	(Ref)			(Ref)			(Ref)		
≥ College	1.716	0.949–3.102	0.074	1.741	0.963–3.148	0.066	1.707	0.946–3.079	0.076
**Age at circumcision**									
Before adulthood	(Ref)			(Ref)			(Ref)		
During adulthood	1.854	1.065–3.229	0.029*	2.041	1.228–3.394	0.006	2.032	1.226–3.367	0.006*
**Reasons for circumcision**									
**Forced by parents**									
Yes	(Ref)								
No	0.647	0.368–1.137	0.130						
**Phimosis or balanitis**									
No				(Ref)					
Yes				1.398	0.861–2.273	0.176			
**Cosmetic reasons or premature ejaculation**									
No							(Ref)		
Yes							1.534	0.859–2.740	0.148
**Skin colour mismatch**									
Yes	(Ref)			(Ref)			(Ref)		
No	2.591	1.623–4.135	< 0.0001*	2.458	1.546–3.909	< 0.0001*	2.604	1.631–4.159	< 0.0001*
**Hypertrophic scarring**									
Yes	(Ref)			(Ref)			(Ref)		
No	2.284	1.393–3.746	0.001*	2.215	1.357–3.616	0.001*	2.226	1.363–3.635	0.001*
**Excessive shortening of prepuce**									
Yes	(Ref)			(Ref)			(Ref)		
No	3.144	1.447–6.836	0.004*	3.209	1.476–6.975	0.003*	3.233	1.490–7.016	0.003*

*OR* odds ratio, *CI* confidence interval.

*P < 0.05.

## Discussion

Male circumcision is a common procedure worldwide, especially in Western Europe, North America, and the Middle East, owing to cultural and religious reasons. Men in these areas usually undergo circumcision as infants; this practice is known as early infant male circumcision. Their parents, rather than the patients themselves, have the autonomy to decide whether circumcision should be performed. However, the prevalence of early infant male circumcision is low in Eastern Asia; for example, it has been reported that the prevalence of early infant male circumcision is less than 3% in China^5^. Many Eastern Asian men undergo circumcision during childhood, adolescence, or adulthood for various reasons. Children and adolescents may be asked to undergo circumcision by their parents, and they may not be able to understand why they need this surgery. However, adolescents and adults might actively seek circumcision because of phimosis or balanitis. Moreover, some may feel that a “cut penis” looks better (cosmetic reasons), and some believe that circumcision can help resolve the problem of premature ejaculation^6^. We hypothesised that men with varying reasons for circumcision would have varying expectations of the circumcision outcome, thereby further influencing their satisfaction with the procedure.

Based on univariate analysis, we found that satisfied participants were significantly more likely to undergo circumcision due to phimosis or balanitis than unsatisfied participants. Furthermore, the participants who underwent circumcision for cosmetic reasons or premature ejaculation were more likely to be unsatisfied. These findings are compatible with those of previous studies. Pathological conditions of the penis or prepuce, such as phimosis and balanitis, have a tremendous impact on the patient’s quality of life. Discomfort during erection, dyspareunia and recurrent genital infection greatly affect sexual activity. Circumcision can resolve these conditions, thus resulting in high satisfaction rates for these patients^7–9^. However, circumcision does not increase the penile size, and a meta-analysis revealed that circumcision does not affect premature ejaculation^10^. Therefore, if patients chose to undergo circumcision because of cosmetic reasons or premature ejaculation, then they were at higher risk for disappointment, resulting in lower satisfaction rates.

Moreover, the univariate analysis indicated that involuntary male circumcision (as a result of the forceful insistence of the parents) was significantly associated with dissatisfaction. Similarly, according to the univariate and multivariate analyses, circumcision during adulthood was significantly associated with participant satisfaction. Therefore, patient autonomy may have an important role in the level of satisfaction experienced by patients. Some studies performed in Africa have shown that adults were satisfied with the outcomes of circumcision and had good acceptance levels^11,12^. Another study revealed that adolescents experienced better satisfaction than children (98.70% versus 94.70%; *P* = 0.035); however, the satisfaction of adolescents was similar to that of adults (98.70% versus 100.00%; *P* = 0.071) circumcised using the Chinese Shang Ring^13^. Boys may not be able to understand the reason for or purpose of circumcision, and they may have a painful experience while undergoing circumcision. Negative emotions and post-traumatic stress disorder have been reported after circumcision performed on children^14^. Other possible factors related to the level of satisfaction are difficulties with wound care and surgical complications that affect the outcomes of circumcision. Childhood circumcision might affect some domains of male sexual function, especially premature ejaculation during adulthood, which may affect the level of satisfaction with circumcision after reaching adulthood^15^.

Based on the multivariate analysis, long-term postoperative complaints, especially the presence of skin colour mismatch, hypertrophic scarring, and excessive shortening of the prepuce, were the most significant predictors of participant satisfaction. Changes in masturbation methods and redundant prepuce after circumcision did not have an effect on satisfaction. Fekete et al*.* reported that circumcised adults were unsatisfied with the procedure and underwent surgical revisions, most commonly due to hypertrophic scarring (21.4%) and scar wrinkling (13.3%)^16^. The postoperative cosmetic appearance affects patient satisfaction and can motivate patients to undergo revision surgery, even if they underwent circumcision for non-cosmetic reasons. Therefore, surgical techniques to avoid conspicuous scars and colour mismatch have been proposed to improve patient satisfaction^17^. Excessive shortening of the prepuce after the circumcision was infrequent observed during our study (reported by 9% of the participants). Excessive loss of foreskin after circumcision can cause erectile pain or pain during intercourse, thus affecting the sexual life of patients enormously; therefore, further reconstruction surgery is often warranted^18,19^. However, participants with redundant prepuce after circumcision do not experience dyspareunia, especially if they chose to undergo circumcision because of phimosis or balanitis. Therefore, this outcome does not have a significant effect on participant satisfaction.

Interestingly, 46.5% of the participants in our study reported penile elongation after circumcision, which was a significant finding among satisfied participants. Additionally, 22.9% of the participants reported prolonged ejaculatory latency time. Obviously, circumcision does not change penile length, but a previous study reported that circumcision improved the confidence of males regarding erections^20^, which may improve the subjective perception of penile size and ejaculatory latency time. Some studies showed that circumcision can increase ejaculatory latency time and improve sexual activity and satisfaction^18,21,22^, while others revealed that circumcision increased pain during intercourse, made it difficult to reach orgasm, and decreased satisfaction with the procedure^23–26^. Other studies indicated no difference in ejaculatory latency time before and after circumcision^20,27–29^. A meta-analysis performed in 2018 concluded that circumcision did not influence premature ejaculation^10^. Nevertheless, we speculated that circumcision might prolong ejaculatory latency time for a specific group of patients who previously had normal ejaculatory latency time. Further studies are warranted to determine the characteristics of these patients with prolonged ejaculatory latency time after circumcision.

This study had several limitations. First, this study was based on an online survey of circumcised men in Taiwan. It was not a randomized sampling from the general population. On the other hand, the circumcised adult Taiwanese males could freely access the online questionnaire, and therefore, potential selection bias may existed. The circumcised status was reported by participants themselves rather than physical examination by urologists, which might have led to some errors; however, this condition usually occurred when the participants received neonatal circumcision. The prevalence of neonatal circumcision in Taiwan was quite low, ranging from 0% to 1.4%^30^. Moreover, we excluded those who were not sure about their circumcised status from the present study. Second, circumcision was performed by several urologists; therefore, operator-associated factors and factors associated with the patient-physician relationship, which were difficult to evaluate, may have affected patient satisfaction. Third, the long-term complaints associated with circumcision were based on subjective reports by the participants rather than objective evaluations. Moreover, we did not ask the participants to quantify their ejaculatory latency time and penile size because this was difficult to accomplish through an online survey. We also did not include dyspareunia as one of the long-term complaints in our questionnaire. However, the subjective perceptions of these long-term conditions, rather than objective measurements, influence participant satisfaction. Fourth, we evaluated the satisfaction experienced by the participants, but not that experienced by their partners. A partner’s point of view regarding circumcision might be an important part of the patient’s decision-making, and several studies indicated that women prefer circumcised penises^6,31^. Nevertheless, the results of the present study remain noteworthy. To our knowledge, this is the first study to evaluate the effects of the reasons for circumcision on patient satisfaction. We also showed that circumcision during adulthood, because of patient autonomy, could improve patient satisfaction and that factors affecting the postoperative cosmetic appearance of the penis, especially hypertrophic scarring, and skin colour mismatch, remain important to patient satisfaction. This information can help improve effective preoperative patient counselling.

In conclusion, two-thirds of the circumcised men involved in this study felt satisfied with the procedure, especially those who underwent circumcision for phimosis or balanitis. Circumcision during adulthood, based on patient autonomy, was predictive of satisfaction. Participants without long-term complaints, such as skin colour mismatch, hypertrophic scarring, and excessive shortening of the prepuce, also reported satisfaction with circumcision. Proper preoperative patient selection and the prevention of postoperative complaints could improve patient satisfaction with circumcision.

## Materials and methods

An online questionnaire was designed and written in the traditional Chinese language to investigate the relationship between the reasons for circumcision and participants’ satisfaction. The online questionnaire was administered via Google Forms, a commercial software application for creating customised survey questionnaires. The online questionnaire was provided with a hyperlink via Facebook. Adult Taiwanese male Facebook users aged between 20 and 40 years who have received circumcision prior to the investigation were invited to participate in the study. Men answered the question “Have you ever received a circumcision procedure?” with yes were deemed as circumcised. Those who were not sure about their circumcised condition were excluded from the present study. The online questionnaire was available from March 7th to 11th, 2020, and was filtered through an internet address check protocol to prevent double filling. According to the Taiwan Network Information Center, the social media use rate of males aged 20–40 in Taiwan was 96.1% to 98.8%, and Facebook users accounted for 94.2% of all users, which equalled 3,260,334 males^32^. Moreover, based on the previous study, the prevalence of circumcision was 8.7% for Taiwanese boys aged 13 years^30^. We estimated the prevalence of circumcision among Taiwanese males aged 20–40 would be 10–15%, which equalled 326,034 to 489,050 males. Based on sample size estimation by G Power software 3.1, a sample size of 376 participants would result in a power between 0.5 to 0.6 in logistic regression analysis.

Demographic data of the participants, including age at circumcision, current age, level of education, and sexual experience, were collected. We asked the participants to categorise their reasons for circumcision and allowed them to choose one or more of the following responses: forced by their parents; phimosis and balanitis; cosmetic reasons and premature ejaculation; or others. We also inquired about the presence of the following long-term conditions after circumcision: subjective changes in penile length and ejaculatory latency time; skin colour mismatch; change in masturbation methods; hypertrophic scarring; excessive shortening of the prepuce, and redundant prepuce. We used a visual analogue scale with scores ranging from 0 to 10 to evaluate patient satisfaction with circumcision. Patients with visual analogue scale scores ≥6 were defined as satisfied participants.

Statistical analyses were performed using IBM SPSS Statistics for Mac (version 24; IBM Corp., Armonk, NY, USA). All data are expressed as numbers (percentages). Pearson’s chi-square test was used to compare the differences between the satisfied and unsatisfied participants, including demographic data, reasons for circumcision, and the presence of long-term complaints. All the perioperative factors were analysed using univariate logistic regression with a single predictor to determine their odds ratio and 95% confidence intervals to predict patient satisfaction. Factors that showed a significant difference in the univariate logistic regression test were included in multivariate logistic regression, including age at circumcision, reasons for circumcision, and presence of certain long-term complaints. The p value of the level of education to predict participants’ satisfaction was 0.051, which was close to the definition of significance. There was a significant difference in the level of education between satisfied and unsatisfied participants in the chi-square test; therefore, we also enrolled this factor in the multivariate logistic regression. Multivariate logistic regression was conducted to determine the independence of predictive factors of participants’ satisfaction with circumcision. We tested the binomial assumption for logistic regression. The odds ratio between satisfied and unsatisfied patients was 1.994 and p value was less than 0.0001. For the overall model, Hosmer and Lemeshow test was used for the goodness-of-fit test, showing that the chi-square test was 9.959, the degree of freedom was 7, and p value was 0.191. Moreover, we also evaluated the individual reason for circumcision as a predictor of participants’ satisfaction after controlling level of education, age at circumcision, and long-term complaints with a multivariate logistic regression test (Table 3). Statistical significance was set as a two-sided *P* < 0.05.

### Ethics statement

The study protocol was approved by the institutional review board (IRB) of Taipei City Hospital (IRB no. TCHIRB-11012006-E). The need for informed consent was waived by the IRB because all participants were anonymous. All procedures were performed in accordance with the principles outlined by the ethics committee and the Declaration of Helsinki.

## Supplementary Information

Supplementary Information.
