# Supplementary material for: Factors influencing satisfaction with male circumcision in Taiwan
Source: Sci Rep. 2023 Feb 9;13:2313. doi: 10.1038/s41598-022-20140-8 (PMC9911792; doi:10.1038/s41598-022-20140-8)
Supplement: Supplementary file 1 — Supplementary Information. [file 41598_2022_20140_MOESM1_ESM.pdf]

We want to know what you think about circumcision!

Hello everyone, this is Dr. Cheng

I have shared with you a lot of information about male circumcision and related health education videos, which have received many responses and solved most people's questions about foreskin; however, there are still many private messages. It seems that there are still some doubts, so I decided to investigate everyone's experiences and thoughts on circumcision. The results of the survey will be used to analyze the data in the future and provide medical research and clinical reference. Later, the results of the survey and research will be announced and shared with everyone, so that everyone can have a more complete understanding of circumcision.

Whether you have cut or not, want to cut, or don't want to cut, you are welcome to enter and fill in our questionnaire.

*Required

Informed consent

Before filling out the questionnaire, please read the relevant risks in detail, and continue to fill in the questionnaire if you agree with the following:

The purpose of this questionnaire is to study adult men's views on circumcision surgery, their acceptance of the surgery, and the impact before surgery on their decision. Causes and correlations with postoperative satisfaction. And to explore the influencing factors of male satisfaction and acceptance of circumcision; including the promoting and hindering factors of circumcision in males who have not received surgery, preoperative reasons for circumcision males and their influence on postoperative satisfaction-related factors; including genital hygiene, changes in the appearance and beauty of the foreskin, the influence of post-operative sexual habits and sexual function, etc. The secondary purpose is to study the factors of the patients themselves, including age, education level, sexual habits and frequency and other factors and the correlation of surgical satisfaction and acceptance.

The chances of possible side effects from filling out this questionnaire are extremely low, including psychological discomfort when filling out the questionnaire. The possible risks of filling in the information include the risks of information leakage from social networking sites and personal information leakage. Please evaluate the risks by yourself before deciding whether to fill in this questionnaire. If you do not agree with this research, you can stop filling in the questionnaire, and the relevant information will be cleared with help.

The information and research results obtained from this questionnaire are for research purposes only. The storage period is 5 years, and only the project host and relevant

researchers in the project will have access to these materials during the storage period. Data will be destroyed immediately after the study.

The findings of this study will be helpful in pre-circumcision counseling and health education.

If you agree and accept the above risks to participate in this research, please continue to assist in filling out this questionnaire

. If you do not agree, you can stop filling in the questionnaire. Thank you for your patience in participating

(Note 1) https://www.ctwant.com/article /110618

Do you agree to participate in this research questionnaire*

☐ Agree and accept the above risks to participate in this research, continue to fill out this questionnaire

☐ If you do not agree, stop filling in the questionnaire

Basic information

1. Your education level*

☐ Below junior high school

☐ Senior High school

☐ The University

☐ Institute and above

2. Your age (___ years old)*

3. First Sexual Experience (_____ years old)*

4. Sexual activity (including intercourse and masturbation)*

☐ More than once a day

☐ Once a week to once a day

☐ Once every two weeks to once a week

☐ Once a month to every two weeks

☐ Almost none (including asexual experience)

5. Have you ever received a circumcision surgical procedure?*

☐ Yes, I do

☐ No, but I want to cut

☐ No, I don't want to cut

If you answered "Yes" in question 5, please answer questions 6 ~ 20

6. Why are you circumcised? (multiple choice)*

☐ Phimosis
☐ Requested by parents
☐ Hygiene and cleanliness
☐ Recurrent inflammation of the glans
☐ Cosmetics
☐ Other: ______________

7. Before the surgery, how painful did you imagine the circumcision surgery would be? (1~10 points; 1 point means no pain at all, 10 points means the most pain)*

____

8. When did you have the surgery? (___ years old)*

9. How long have you been circumcised since filling out the questionnaire now? ______ years*

10. What impressed you the most about circumcision*

☐ Forgotten
☐ Feeling of needle puncture
☐ It hurts at the end of the procedure
☐ Very Nervous
☐ Very cold
☐ Other: ______________

What was the actual pain for a week after surgery? (1-10 points; 1 point means no pain at all, 10 points means the most pain)*

____

12. What was the most unbearable thing for me within a week after the operation? (check)*

☐ Pain when changing dressings or getting an erection
☐ Swelling and bleeding wounds worry me
☐ The wound can't touch the water, can't take a bath
☐ It is difficult to take care of dressing changes, and unable to bandage
☐ To suspend sex
☐ Shaved pubic hair is itchy
☐ Other: _____________

Do you think your penis looks bigger after the wound is fully healed?*

☐ No difference
☐ Looks Bigger
☐ Looks smaller

14. After the wound has fully healed, do you feel you have become more durable?*

☐ No difference

☐ Become more durable

☐ Become less durable

15. After the wound has completely healed, have you felt a change in your pleasure in sex or masturbation?*

☐ No difference

☐ Become more enjoyable

☐ Become more unpleasant

16. After the wound is completely healed, is there any problem with your foreskin? (check)*

☐ None

☐ Penis skin color mismatch

☐ Surgical suture hypertrophy

☐ Masturbation or sexual behavior needs to change

☐ The foreskin is too short, making it uncomfortable to have an erection

☐ The foreskin still wraps the glans

☐ Other:

17. How satisfied are you with the surgery? (1~10 points; 1 point means very dissatisfied; 10 points means very satisfied)

18. Do you feel worth it or regret it? (1~10 points; 1 point means very regretful, 10 points means very worthwhile)*

19. If you could start all over again, would you choose to be circumcised?*

☐ Yes, I do

☐ No, I won't

20. If someone asked you, would you advise someone to be circumcised?*

☐ Yes, I do

☐ No, I won't

Please answer questions 21 ~ 30 if you fill in "No, but I want to cut / I don't want to cut" in question 5

21. Why do you want to be circumcised? (multiple choice)*

☐ Phimosis

☐ Requested by parents

☐ Hygiene and cleanliness

☐ Recurrent inflammation of the glans

☐ Cosmetics

☐ Other: ______________

22. How painful would you imagine circumcision surgery would be? (1~10 points; 1 point means no pain at all, 10 points means the most pain)*

____

23. How long have you considered circumcision? _____ year*

24. Why haven't you made up your mind to be circumcised? (check)*

- ☐ Fear of pain after surgery
- ☐ Fear of being laughed at by others
- ☐ I don't know how to take care of the wound
- ☐ Not having sex while waiting for the wound to heal
- ☐ Seeing other people's unpleasant experiences
- ☐ Can't find the right time
- ☐ I don't know whom to turn to for circumcision
- ☐ Afraid of the ugly wound after the procedure
- ☐ No money
- ☐ Other:

25. Under what circumstances would you make up your mind to undergo circumcision? (check)*

- ☐ Appropriate health education information is available on the Internet
- ☐ Doctor's detailed explanation
- ☐ Strong request from partner or parent
- ☐ Recommendation from a colleague or friend
- ☐ Other:

26. Do you think the penis will look bigger after circumcision?*

- ☐ No difference
- ☐ Looks Bigger
- ☐ Looks smaller

27. Do you think it will last longer after circumcision?*

- ☐ No difference
- ☐ Become more durable
- ☐ Become less durable

28. Do you think the pleasure of sex or masturbation will change if you are circumcised?*

- ☐ No difference
- ☐ Become more enjoyable
- ☐ Become more unpleasant

29. Do you need circumcision under any of the following conditions? (check)*

- ☐ None
- ☐ Phimosis, foreskin too tight

- [ ] Hygiene and cleanliness
- [ ] Recurrent inflammation of the glans
- [ ] Hope it lasts longer
- [ ] Want to make the penis look bigger
- [ ] Other: _________

30. If you were circumcised, what would you worry about? (check)*

- [ ] Fear of pain after surgery
- [ ] Fear of being laughed at by others
- [ ] I don't know how to take care of the wound
- [ ] Concerned about the risks of anesthesia
- [ ] Worry about complications such as bleeding
- [ ] Seeing other people's unpleasant experiences
- [ ] Fear of affecting erectile function
- [ ] Fear of decreased pleasure during sex after mutilation
- [ ] Afraid of being ugly after the procedure
- [ ] Can't find the right time
- [ ] I don't know whom to turn to for circumcision
